# Discussions on the quality of antibodies are no reason to ban animal immunization

**DOI:** 10.15252/embr.202051761

**Published:** 2020-11-12

**Authors:** René Custers, Jan Steyaert

**Affiliations:** ^1^ VIB (Flanders Institute for Biotechnology) Ghent Belgium; ^2^ Structural Biology Brussels Vrije Universiteit Brussel (VUB) Brussels Belgium; ^3^ VIB‐VUB Center for Structural Biology VIB Brussels Belgium

**Keywords:** S&S: Economics & Business, Methods & Resources, Chemical Biology

## Abstract

Debates about the source of antibodies and their use are confusing two different issues. A ban on life immunization would have no repercussions on the quality of antibodies.

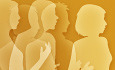

There is an ongoing debate on how antibodies are being generated, produced and used (Gray, 2020; Marx, 2020). Or rather, there are two debates, which are not necessarily related to each other. The first one concerns the quality of antibodies used in scientific research and the repercussions for the validity of results (Bradbury & Pluckthun, 2015). The second debate is about the use of animals to generate and produce antibodies. Although these are two different issues, we observe that the debates have become entangled with arguments for one topic incorrectly being used to motivate the other and *vice versa*. This is not helpful, and we should disentangle the knot.

Polyclonal antibodies are being criticized because they suffer from cross‐reactivity, high background and batch‐to‐batch variation (Bradbury & Pluckthun, 2015). Monoclonal antibodies produced from hybridomas are criticized because they often lack specificity owing to genetic heterogeneity introduced during hybridoma generation that impairs the quality of the monoclonals (Bradbury *et al*, 2018). These are valid criticisms and producing antibodies in a recombinant manner will, indeed, help to improve quality and specificity. But a mediocre antibody will remain a mediocre antibody, no matter how it is produced. Recombinant methods will just produce a mediocre antibody more consistently.

Getting a good antibody is not easy and much depends on the nature and complexity of the antigen. And low‐quality antibodies are often the result of poor screening, poor quality control, incomplete characterization and the lack of international standards. Nevertheless, the technologies to ensure good selection and to guarantee consistent quality are much more advanced than a decade ago, and scientists and antibody producers should implement these to deliver high‐quality antibodies. Whether antibodies are generated by animal immunization or from naïve or synthetic antibody libraries is less relevant; they can all be produced recombinantly, and screening and characterization are needed in all cases to determine quality, and if the antibody is fit for purpose.

But criticisms on the quality of many antibodies and pleas for switching to recombinant production of antibodies cannot be mixed up with a call to ban animal immunization. The EU Reference Laboratory for Alternatives to Animal Testing (EURL ECVAM) recently published a recommendation to stop using animals for generating and producing antibodies for scientific, diagnostic and even therapeutic applications (EURL ECVAM, 2020). This recommendation is mainly supported by scientists who seem to be biased towards synthetic antibody technology for various reasons. Their main argument is that antibodies derived from naïve or synthetic libraries are a valid (and exclusive) alternative. But are they?

One can certainly select antibodies from non‐immune libraries, and, depending on the antigen and the type of application, these antibodies can be fit for purpose. In fact, a few of such antibodies have made it to the market as therapeutics, Adalimumab (Humira®) being a well‐known example. But up to now, the vast majority of antibodies continues to come from animal immunization (Lu *et al*, 2020). And there is a good reason for that. It is generally possible to generate a few positive hits in a naïve/synthetic library; and the more diverse the library, the more hits one is likely to get. But many decades of experience with immunization of animals—especially when they are outbred—shows that they generate larger amounts of antibodies with superior properties. And the more complex your antigen is, the more the balance swings towards animal immunization if you want to have a guarantee for success.

There are different factors at work here. First, the immune system of mammals has evolved over millions of years to efficiently produce excellent antibodies against a very diverse range of antigens. Second, presenting the antigen multiple times in its desired (native) conformation to the animal immune system exploits the natural maturation process to fine‐tune the immune response against particular qualities. Another factor is that *in vivo* maturation seems to select against negative properties such as self‐recognition and aggregation. It also helps to select for important properties that go beyond mere molecular recognition (Jain *et al*, 2017). In industrial parlance, antibodies from animal immunization are more “developable” and have favourable biophysical properties (Lonberg, 2005). Indeed, the failure rate for antibodies selected from naïve or synthetic libraries is significantly higher.

Of course, the properties of synthetic antibodies selected from non‐immune libraries can be further matured *in vitro*, for example by light chain shuffling or targeted mutagenesis of the complementarity determining region (CDR). While this method has become more sophisticated over the years, it remains a very complex and iterative process without guarantee that it produces a high‐quality antibody.

Antibodies are an ever more important tool in scientific research and a growing area in human and veterinary therapeutics. Major therapeutic breakthroughs in immunology and oncology in the past decades are based on antibodies (Lu *et al*, 2020). The vast majority of these therapeutic antibodies were derived from animals. An identical picture appears when you look at the antibodies in fast‐track development to combat the current COVID‐19 crisis: again, the vast majority are either derived from patients or from animal immunizations. The same holds true for antibodies that are used in diagnostics and epidemiologic studies for COVID‐19.

It is for that reason that we need the tools and methods that guarantee antibodies of the highest quality and provide the best chance for success. The COVID‐19 pandemic is only one illustration of this need. If we block access to these tools, both scientific research and society at large will be negatively impacted. We therefore should not limit ourselves to naïve and synthetic libraries. Animal immunization remains an inevitable method that needs to stay. But we all agree that these immunizations must be performed under best practice to further reduce the harm to animals.

## Conflict of interest

Jan Steyaert runs Nanobodies4Instruct, a flagship platform of Instruct‐ERIC that is part of the European Strategy Forum on Research Infrastructures (ESFRI). This facility focusses on the discovery of Nanobodies to be used in Structural Biology. He is also founder and senior advisor of ConfoTherapeutics.
